# Tumours, Innocent and Malignant

**Published:** 1894-06

**Authors:** 


*Tumours, Innocent and Malignant.* By J. BLAND SUTTON. Pp. xvi., 511. London: CASSELL AND COMPANY, LIMITED. 1893.

It is always a pleasant task to review a book written by Mr. Bland Sutton, because the originality of his work distinguishes it at once from the mere compilations of so many authors. This book on tumours is no exception to the rule. From the author of *Dermoids and Ovarian Tumours* we expect much when he writes on tumours in general; and as we examine this book our expectations are abundantly realised. Of course much that Mr. Bland Sutton has previously told us is given again, but here every form and variety of tumour is described. By far the greater part of the volume is taken up with the pathology of tumours, but their treatment is also given. The book is like the rest of Mr. Sutton's works, particularly well illustrated, and contains several good coloured plates. Many of the drawings are original, and not a few are from specimens in the Middlesex Hospital Museum. Amongst the facts with regard to tumour pathology which have long been recognised, we come every now and again on information which has only recently been obtained, very frequently by the writer himself. All the recent work which has been done in connection with the pathology of tumours, both in this country and abroad, has been utilised to supply us with a text-book thoroughly up to date.

Perhaps some will regret that the chapter devoted to the cause of tumours is not a long one. We turned to it with much interest, and at first we were almost inclined to regret that it did not go more fully into the subject; but after all, but little is really known about the cause of tumours. Theory after theory arises, lives for a short time, and then almost imperceptibly expires, and observations on which to build a surer foundation of knowledge only slowly—very slowly—accumulate. Hence, perhaps Mr. Sutton has been wise to limit his attention to Cohnheim's interesting theory of the origin of tumours from minute portions of embryonic tissue remaining undeveloped amongst the tissues.

The chapters on "Tubulo-cysts" and "Tubulo-dermoids" are some of the most interesting in the book. They show how dermoids and other cysts may arise in unobliterated remains of fœtal structures. Mr. Sutton still holds the view of Coblenz and Doran, that papillomatous cysts of the ovary arise from remains of the Wolffian body, though Olshausen and Whitridge Williams contend otherwise. In the classification of tumours epithelioma is separated from cancer, which is not regarded as simply a malignant epithelial tumour, but rather as a malignant adenoma, while epithelioma is described as a malignant wart. Implantation cysts, or traumatic dermoids as they have been called, are very fully described in a separate chapter, which from the attention given to iritic and corneal cysts of this nature must be of interest to the ophthalmic surgeon as much as to the general surgeon.

This book is one of which we shall constantly be in need, as rare tumours require elucidation; it is, in fact, an encyclopædia of knowledge, with numerous references to recent original work, a very large proportion of which has been done by Mr. Bland Sutton himself.

